# Multifunctional injectable hydrogel incorporating EGCG-Cu complexes for synergistic antibacterial, immunomodulatory, and osteogenic therapy in periodontitis

**DOI:** 10.1016/j.mtbio.2025.101907

**Published:** 2025-05-28

**Authors:** Yajuan Hu, Wei Xu, Linghan Sun, Xuemin Ma, Peirong Zhou, Chuankai Zhang, Rui Cai, Xia Wang, Hua Yang, Gang Tao, Junliang Chen, Yun He

**Affiliations:** a Luzhou Key Laboratory of Oral & Maxillofacial Reconstruction and Regeneration, The Affiliated Stomatological Hospital, Southwest Medical University, Luzhou, 646000, China; b Department of Oral and Maxillofacial Surgery, The Affiliated Stomatological Hospital, Southwest Medical University, Luzhou, 646000, China; c Institute of Stomatology, Southwest Medical University, Luzhou, 646000, China; d Department of General Dentistry, The Affiliated Stomatological Hospital, Southwest Medical University, Luzhou, 646000, China; e Department of Oral and Maxillofacial Surgery, The Deyang Stomatological Hospital, Deyang, 618000, China

**Keywords:** Periodontitis, Hydrogel, Alveolar bone regeneration, Epigallocatechin gallate (EGCG), Copper ions (Cu^2+^)

## Abstract

Periodontitis is a chronic inflammatory disease characterized by oxidative stress, immune system imbalance, and the progressive destruction of periodontal tissues. Traditional treatment approaches are limited by the incomplete eradication of pathogens, the risk of antibiotic resistance, inadequate control of inflammation and oxidative stress, and restricted tissue regeneration capacity. Therefore, this study proposes an injectable multifunctional Laponite/gelatin hydrogel loaded with epigallocatechin gallate (EGCG)-copper ion (Cu^2+^) complexes as a localized therapy for periodontitis. EGCG exhibits antioxidant, anti-inflammatory, and antimicrobial properties; however, its clinical application is hindered by poor stability and bioavailability. Cu^2+^ coordination enhances EGCG stability and antioxidant capacity while improving its antimicrobial efficacy. Experimental results demonstrate that the Laponite/gelatin hydrogel is adaptable for the localized delivery of EGCG-Cu, with bioactive metal ions such as Li^+^, Mg^2+^, and Si^4+^ contained in Laponite, which promote osteogenesis and periodontal tissue regeneration. *In vitro* and *in vivo* studies confirm that this hydrogel exhibits excellent biocompatibility, effectively inhibits *Porphyromonas gingivalis*, suppresses M1 polarization while promoting M2 polarization, and facilitates periodontal tissue repair. Therefore, this study provides promising insights into a localized therapeutic strategy for periodontitis.

## Highlights

• **Multiple ROS Scavenging Capacity:** Cu^2^+ catalyzes antioxidant reactions, boosting EGCG's ability to scavenge reactive oxygen species (ROS), thereby more effectively alleviating oxidative stress.
• **Injectability and Adaptability:** Its adaptability and self-healing properties enable it to fill moist and complex periodontal defects, providing a stable three-dimensional scaffold that promotes cell adhesion and tissue regeneration.
• **Immunomodulatory Performance:** It effectively inhibits M1 polarization of macrophages and promotes their polarization toward the M2 phenotype.
• **Osteogenic Inductive Function:** Cu^2+^ and Laponite synergistically exert osteogenic induction, facilitating alveolar bone regeneration in the context of periodontal inflammation.
• **Antibacterial Activity：**The Lap-Gel/H-E-Cu hydrogel exhibits strong synergistic antibacterial activity of EGCG and Cu against *Porphyromonas gingivalis* and *Staphylococcus aureus*, showing great potential for periodontitis treatment.

## Graphical abstract

**Figure:**
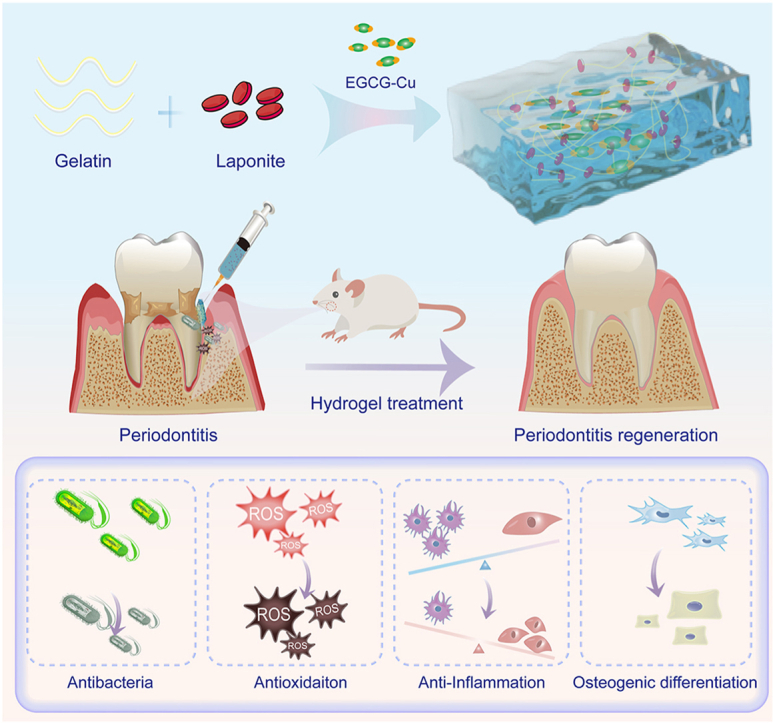

## 1 Introduction

Periodontitis is a chronic inflammatory disease that progressively deteriorates the tooth-supporting structures, including the alveolar bone, periodontal ligament, and gingiva, ultimately resulting in tooth loss [1,2]. The disease is primarily driven by the accumulation of pathogenic microorganisms that proliferate within deep periodontal pockets [2]. In its early stages, microbial stimulation induces the excessive release of inflammatory mediators and reactive oxygen species (ROS) from the periodontal tissues [3,4]. These mediators exacerbate the local inflammatory response, thereby promoting further destruction of periodontal tissues [5,6].

Given the central role of oxidative stress, inflammation, and bacterial infection in disease progression, strategies targeting bacterial eradication ROS scavenging, and inflammation modulation are considered promising therapeutic approaches for periodontitis [7,8]. Macrophages play a critical role in the immune response during periodontitis [9]. They are typically classified into pro-inflammatory M1 and anti-inflammatory M2 phenotypes [10]. During the initial inflammatory phase, M1 macrophages contribute to pathogen clearance and immune defense; however, their persistent activation may exacerbate disease progression, ultimately leading to irreversible periodontal tissue destruction [3,7]. In contrast, M2 macrophages facilitate inflammation resolution, tissue repair, and regeneration through the secretion of anti-inflammatory cytokines [11]. Therefore, suppressing M1 polarization and enhancing M2 polarization have become key therapeutic targets for periodontitis.

However, chronic inflammation is closely associated with persistent bacterial infection. Pathogens residing in periodontal pockets continuously activate the host immune response, sustaining a chronic inflammatory state and exacerbating tissue destruction. Clinically, mechanical debridement combined with antibiotic therapy is effective in reducing periodontal pathogens. However, prolonged antibiotic use presents several challenges, including bacterial resistance, microbiome dysbiosis, and gastrointestinal complications [12,13]. Moreover, conventional antimicrobial treatments exhibit limited efficacy in modulating inflammation and scavenging ROS, both of which are critical in disease progression [14]. Therefore, an effective therapeutic strategy for periodontitis should not only eradicate pathogens but also modulate inflammation and mitigate oxidative stress, thereby creating a favorable microenvironment for periodontal tissue regeneration.

Numerous natural compounds have been widely recognized for their antibacterial, antioxidant, and anti-inflammatory properties, and they have been extensively utilized in the treatment of various diseases, including cancer, malaria, and periodontitis [15,16]. Among these, epigallocatechin-3-gallate (EGCG), a key polyphenol extracted from green tea, has garnered considerable attention due to its potent biological activities, including antimicrobial antioxidant, and anti-inflammatory effects [4,17,18]. However, despite its promising therapeutic properties, the clinical application of EGCG in periodontitis treatment remains challenging due to its poor bioavailability and limited stability [19,20]. Within the oral environment, EGCG undergoes rapid enzymatic degradation, which significantly compromises its therapeutic efficacy [17]. Recent studies have demonstrated that the biological activity and stability of EGCG can be substantially enhanced through coordination with metal ions, which also imparts additional therapeutic advantages [21]. Among these, Cu^2+^, an essential trace element [22], plays a vital role in numerous physiological processes, such as erythropoiesis, energy metabolism, and antioxidant defense [2]. Notably, Cu^2+^ has been implicated in bone healing and remodeling, further underscoring its potential in periodontal regeneration [23]. Additionally, Cu^2+^ exhibits broad-spectrum antimicrobial properties by disrupting bacterial biofilms, impairing bacterial structural integrity, and inducing the denaturation of intracellular enzymes, ultimately leading to bacterial apoptosis [2,23,24]. To achieve the sustained release of EGCG and Cu^2+^ at the site of periodontitis, an EGCG-Cu metal-polyphenol complex has been developed. This complex facilitates the controlled release of both EGCG and Cu^2+^, thereby enhancing antimicrobial efficacy, modulating the inflammatory microenvironment, and promoting alveolar bone regeneration. While the EGCG-Cu complex holds significant therapeutic potential, the development of an optimized drug delivery system with controlled and sustained release properties is essential to maximize its efficacy in periodontitis treatment [25,26].

Hydrogels are widely studied as multifunctional drug delivery platforms due to their ability to provide controlled release of therapeutic agents with antibacterial, antioxidant, and anti-inflammatory properties [13,24,25]. Among them, shear-thinning hydrogels offer particular advantages for periodontal applications: their non-Newtonian behavior allows them to flow under stress and recover rapidly, enabling easy injection into irregular periodontal defects [26,27]. To enhance therapeutic efficacy, we propose an injectable shear-thinning hydrogel incorporating the EGCG-Cu complex for dual delivery of EGCG and Cu^2+^. Gelatin, a biodegradable and biocompatible natural polymer, serves as the hydrogel matrix [28]. Laponite, a layered silicate nanomaterial with high surface area and ion-exchange capacity, further reinforces the hydrogel [29]. Electrostatic interactions between gelatin and Laponite facilitate the formation of stable nanocomposite hydrogels with improved mechanical strength and drug encapsulation efficiency [30]. Importantly, Laponite also contains bioactive ions such as lithium (Li^+^), magnesium (Mg^2+^), and silicon (Si^4+^), which promote osteoblast differentiation, extracellular matrix mineralization, and bone regeneration. These synergistic properties make the Laponite/gelatin-based EGCG-Cu hydrogel a promising platform for periodontal tissue repair and regeneration [28,30].

**Fig. 1:**
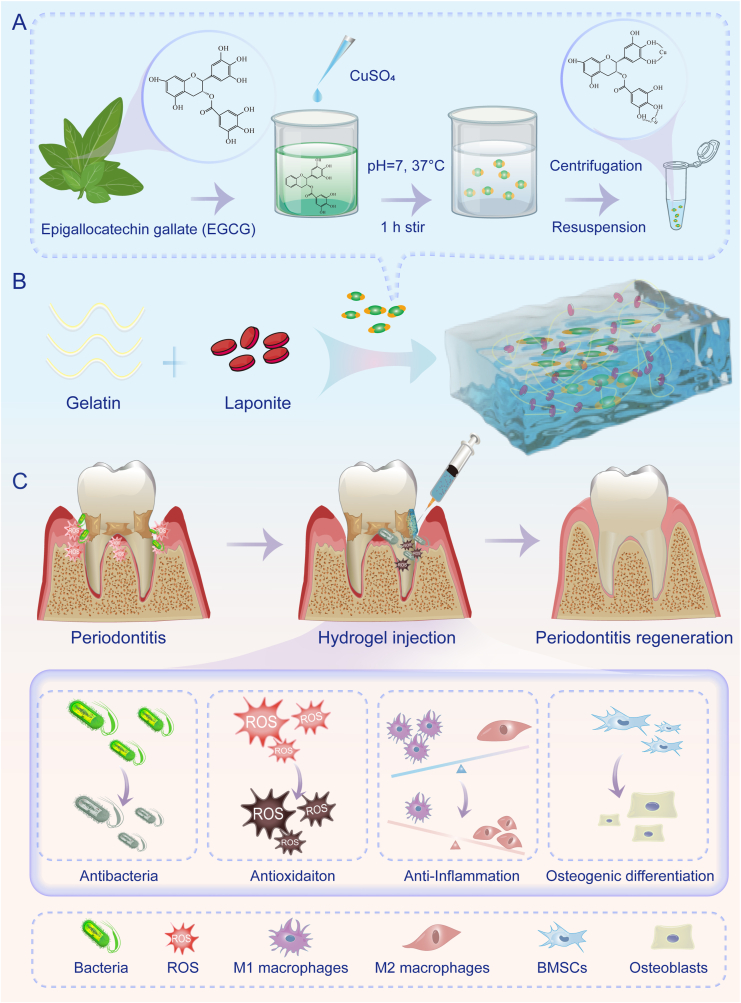
Schematic illustration of the fabrication and application of Lap-Gel/E-Cu hydrogel for periodontitis treatment. (A) Schematic diagram of the synthesis of EGCG-Cu complex. (B) Schematic diagram of the synthesis of Lap-Gel/E-Cu hydrogel. (C) Schematic representation of the antibacterial, ROS-scavenging, immunomodulatory and osteogenesis-promoting functions of Lap-Gel/E-Cu hydrogel in the treatment of periodontitis.

In this study, we present an injectable hydrogel platform encapsulating EGCG-Cu complexes for the treatment of periodontitis. To achieve this, we first synthesized EGCG-Cu complexes (Fig. 1A), which were subsequently incorporated into an injectable, self-healing hydrogel scaffold with shear-thinning properties. This hydrogel, designated as Lap-Gel/E-Cu, was formulated using gelatin and Laponite to ensure optimal bioactivity and structural stability (Fig. 1B). Our experimental data demonstrate that the hydrogel exhibits excellent biocompatibility, inducing no cytotoxicity or adverse effects *in vitro* or *in vivo*. In vitro studies confirmed that this hydrogel effectively inhibits bacterial growth, inhibited M1 macrophage polarization while enhancing M2 macrophage polarization, and enhances osteogenic differentiation, collectively facilitating periodontal tissue regeneration. Furthermore, its therapeutic efficacy was validated *in vivo* using a Sprague-Dawley (SD) rat model of periodontitis, where it significantly improved periodontal healing outcomes. In conclusion, this study presents Lap-Gel/E-Cu hydrogel as a comprehensive and innovative strategy for addressing the multifaceted challenges of periodontitis. By integrating antimicrobial, antioxidant, immunomodulatory, and osteogenic properties, this hydrogel offers a promising approach for localized adjunctive treatment of periodontitis (Fig. 1C).

## 2 Materials and methods

### 2.1 Materials

Epigallocatechin-3-gallate (EGCG, 98 %) was purchased from Shanghai Macklin Biochemical Technology Co., Ltd. (Shanghai, China). CuSO_4_·5H_2_O (98.0 %) and NaHCO_3_ were purchased from Shanghai Aladdin Biochemical Technology Co., Ltd. (Shanghai, China). Hydrogen peroxide (H_2_O_2_, 30 wt%) and poly(sodium 4-styrenesulfonate) (PSS, MW ∼70,000) were provided by Tianjin Guangfu Fine Chemical Research Institute (Tianjin, China). Phosphate-buffered saline (PBS), anhydrous dimethyl sulfoxide (DMSO, 99.9 % purity), α-modified Eagle medium (α-MEM, VivaCell, China), Dulbecco's Modified Eagle's Medium (DMEM), fetal bovine serum (FBS), Tween 20, lipopolysaccharide (LPS), and phorbol 12-myristate 13-acetate (PMA) were sourced from Solarbio Science & Technology Co., Ltd. (Beijing, China). The 1,1-diphenyl-2-picrylhydrazyl (DPPH) free radical (product number: D4313) was acquired from Tokyo Chemical Industry Co., Ltd. Transwell BD Matrigel was sourced from Corning Incorporated (New York, USA). Antibodies for iNOS, CD206, RUNX2, and OCN were acquired from Abcam (Cambridge, UK), while PCNA was obtained from Santa Cruz Biotechnology Inc. (CA, USA). All chemicals were used without further purification.

### 2.2 Synthesis and characterization of EGCG-Cu coordination compound

1 mL of EGCG (0.2 mM) and 1 mL of CuSO_4_·5H_2_O (0.4 mM) were successively added to 8 mL of deionized water (DI water). Subsequently, an alkaline solution of NaHCO_3_ (pH 8.3) was added to adjust the pH of the suspension, facilitating compound formation, and the mixture was stirred for 10 min at room temperature. The resulting precipitates were washed to remove excess EGCG and Cu^2+^. The washing process was repeated three times, resulting in a 10 mL suspension of EGCG-Cu coordination compounds. The elemental composition, morphologies, and distribution of EGCG-Cu were detected using EDS combined with TEM. XPS was utilized to analyze the copper content and variations in binding energy. Fourier transform infrared (FTIR) spectra were performed to analyze the main functional groups. UV–vis absorption spectra were measured at 25 °C.

### 2.3 Preparation and characterization of injectable Laponite/gelatin hydrogel loaded with EGCG-Cu (Lap-Gel/E-Cu)

The Lap-Gel/E-Cu injectable hydrogel was prepared using a one-pot synthesis method. First, 0.6 g of gelatin (Aladdin, China) was dissolved in 10 mL of deionized (DI) water at 45 °C, stirring until the gelatin particles were completely dissolved. Next, different volumes of EGCG-Cu suspension (0 mL, 1 mL, 5 mL, and 10 mL) were separately mixed with 10 mL of the gelatin solution. Then, 0.6 g of Laponite (BYK, Germany) was added to each mixture. Finally, the total volume of each solution was adjusted to 20 mL, forming a uniform hydrogel with varying EGCG-Cu concentrations (Lap-Gel, Lap-Gel/L-E-Cu, Lap-Gel/M-E-Cu, Lap-Gel/H-E-Cu). To assess the release behavior of active agents, the Lap-Gel/H-E-Cu hydrogel was immersed in phosphate-buffered saline (PBS, pH 7.4) and incubated under physiological conditions. At predetermined time intervals, aliquots of the supernatant were collected and replaced with an equal volume of fresh PBS to maintain sink conditions. The concentration of released EGCG was determined using a microplate reader (Tecan Infinite M200-Pro, China) at an absorbance wavelength of 274 nm. The amount of released Cu^2+^ was quantified using Inductively Coupled Plasma Optical Emission Spectrometry (ICP-OES, Agilent 5110, USA).

After freeze-drying, the hydrogel morphology was analyzed using a scanning electron microscope (SEM, Zeiss Sigma300, Germany). The surface elemental composition and distribution were analyzed using energy-dispersive spectroscopy (EDS) in conjunction with SEM. Additionally, FTIR spectroscopy was conducted in transmission mode using the KBr technique, covering a spectral range of 2000 to 400 cm^−1^ for all four hydrogel formulations.

To assess the degradation behavior of the hydrogel under physiological-like conditions, samples (n = 3) were incubated in phosphate-buffered saline (PBS, pH 7.4) at 37 °C. At predetermined time points (days 1, 3, 5, and 7), the hydrogels were carefully removed, rinsed with deionized water, lyophilized, and weighed. The percentage of remaining mass was calculated using the following formula:

$$\text{Mass}\;\text{remaining}\;\left(\%\right)=\left(\text{Wt}\;/\;W0\right)\times 100\%\;$$

where W0 is the initial dry weight, and Wt is the dry weight at the corresponding time point.

### 2.4 Injection force of hydrogels

The injectability of the hydrogel was assessed using a universal mechanical testing machine (Instron Model 5965, USA). Briefly, the hydrogel was loaded into 5 mL plastic syringes and extruded through clinical-grade medical needles (inner diameter: 0.45 mm; 26G) at a controlled speed. The syringe plunger was compressed by an upper platen, while the syringe housing or catheter was firmly held in a lower tensile grip to maintain stability throughout the test. The injection rate was regulated by adjusting the crosshead speed of the compression platen to obtain the desired flow rates. The force required to depress the plunger was measured using a 50 N load cell. A manual injection demonstration was also conducted to verify the practical injectability under clinical-like conditions.

### 2.5 Antimicrobial activity of the hydrogels *in vitro*

The antimicrobial activity of the different hydrogels was evaluated by colony count method using *Staphylococcus aureus* (*S. aureus*) and *Porphyromonas gingivalis* (*P. gingivalis*). Briefly, *S. aureus* was cultured overnight in LB broth at 37 °C with shaking at 150 rpm, while *P. gingivalis* was grown overnight in BHI broth supplemented with yeast extract, hemin, and vitamin K at 37 °C under anaerobic conditions. The bacterial suspensions were collected, centrifuged at 1500 rpm for 15 min, washed three times with PBS, and then resuspended in fresh culture medium to a concentration of 1 × 10^7^ CFU/mL. Subsequently, 4 mL of the diluted bacterial suspensions were respectively dropped onto 2 mL sterilized DI water, Lap-Gel, Lap-Gel/L-E-Cu, Lap-Gel/M-E-Cu, and Lap-Gel/H-E-Cu. After a predetermined time of incubation (6 h for *S. aureus* and 24 h for *P. gingivalis*), the bacterial suspensions were collected. A 20-fold serial dilution was performed, and colony-forming units were counted using agar plates (LB agar plate for *S. aureus*; BHI blood agar plate for *P. gingivalis*) following appropriate incubation conditions. Bacterial viability was assessed using the LIVE/DEAD® BacLight™ Bacterial Viability Kit according to the manufacturer's instructions. Live bacteria were stained green with the SYTO 9 fluorescence probe, while dead bacteria were stained red with propidium iodide (PI). *S. aureus* (6 h co-cultured with the DI water, Lap-Gel, Lap-Gel/L-E-Cu, Lap-Gel/M-E-Cu and Lap-Gel/H-E-Cu) and *P. gingivalis* (24 h co-cultured with the DI water, Lap-Gel, Lap-Gel/L-E-Cu, Lap-Gel/M-E-Cu and Lap-Gel/H-E-Cu) were collected and went through wash centrifugation process (6000 rpm, 10 min) 3 times. After co-culture with SYTO 9-PI for 15 min at room temperature in the dark, 5 μL of each stained bacterial suspension was trapped between a slide and a coverslip and observed in a fluorescence microscope. Green fluorescent bacteria were captured using a FITC filter (λex/λem = 488/500 nm), while red fluorescent-labeled cells were visualized using a TRICT filter (λex/λem = 550/600 nm).

### 2.6 Cell culture of RAW264.7 and BMSCs

Mouse monocyte-macrophage leukemia cells (RAW264.7) were sourced from the Cell Bank of the Chinese Academy of Sciences (Shanghai, China). They were cultured in Dulbecco's Modified Eagle Medium with High Glucose (DMEM, Gibco, USA), supplemented with 10 % fetal bovine serum (FBS, Gibco, USA) and 1 % penicillin-streptomycin (Beyotime, China), and incubated at 37 °C in a 5 % CO_2_ atmosphere.

To isolate bone marrow mesenchymal stem cells (BMSCs), four-week-old female SD rats were euthanized via cervical dislocation and sterilized in 75 % ethanol for 10 min. Bone marrow was extracted under sterile conditions and immersed in α-MEM supplemented with 10 % fetal bovine serum and 1 % penicillin-streptomycin. The cells were cultured at 37 °C in a 5 % CO_2_ incubator, with the culture medium refreshed every two days. Once the cells reached 60–70 % confluency, they were subcultured for further use.

### 2.7 The blood compatibility and cytocompatibility testing

Fresh blood, which was collected from the heart of four weeks old New Zealand rabbit, anticoagulated (blood: 3.8 % sodium citrate = 9:1), and diluted with 0.9 % physiological saline (blood: saline = 4:5, v/v) for the hemolysis test. 10 mL of DI water, Lap-Gel, Lap-Gel/L-E-Cu, Lap-Gel/M-E-Cu and Lap-Gel/H-E-Cu extracts were incubated in a shaking water bath at 37 °C for 30 min. Then, 200 μL of diluted blood was added, thoroughly mixed, and maintained at 37 °C for 60 min. The supernatant was collected by centrifugation at 3000 rpm for 5 min, and the optical density (OD) was measured using a UV–visible spectrophotometer at 545 nm. Negative and positive controls were set up by mixing 200 μL of diluted blood with 10 mL of either 0.9 % NaCl solution or 1 % Triton X-100 solution, respectively. The hemolysis ratio was determined using the following equation:

$$\text{Hemolysis}\;\text{ratio}\;\left(\%\right)=\left[\left({\text{OD}}_{\text{test}}-{\text{OD}}_{\text{nc}}\right)/\left({\text{OD}}_{\text{pc}}-{\text{OD}}_{\text{nc}}\right)\right]\times 100\%$$

where OD_test_, OD_pc,_ and OD_nc_ represent the optical density values of the test sample, positive control, and negative control, respectively.

Cytocompatibility was assessed through live/dead cell staining and the Cell Counting Kit-8 (CCK-8) assay. Hydrogels were immersed in a complete medium, and the obtained extracts were collected for further analysis. BMSCs and RAW264.7 cells were seeded in 96-well plates and cultured for 24 h. The culture medium was then replaced with the collected hydrogel extracts, and cells were incubated for 1, 3, and 5 days. For the CCK-8 assay, 10 μL of CCK-8 solution was added to each well and incubated at 37 °C for 2 h. The OD value at 450 nm was measured using a microplate reader (TECAN Infinite M200Pro, China). Live/dead staining was performed using an AM/PI double staining kit. Stained cells were examined using a fluorescence microscope (DMI8, Leica, Germany).

### 2.8 Antioxidant activity test

The antioxidant activity of the Lap-Gel hydrogel loaded with EGCG-Cu was assessed by measuring its ability to scavenge DPPH free radicals. The reduction in DPPH radicals was monitored by the change in color from purple to yellow, and the antioxidant activity was quantified by measuring the absorbance at 517 nm. Approximately 5 mL of hydrogel was dissolved in 10 mL of DI water and centrifuged after shaking for 4 h. The DI water group served as the control, while the group with added vitamin C solution (0.5 mg/mL) was used as the positive group. The supernatant was then subjected to further testing. About 2 mL of 0.1 mM DPPH methanol solution was mixed with 2.5 mL of hydrogel supernatant and reacted in the dark for 30 min. The absorbance of the DPPH assay solution at 517 nm was measured using an ultraviolet spectrophotometer (Shimadzu, UV-2007, Japan). The test tube was shaken to ensure uniformity of the solution before measurement. The DPPH scavenging activity (R (%)) was calculated using the formula:

$$R\;\left(\%\right)=\left(A_{2}-A_{1}\right)/A_{0}$$

where A_0_ is the absorbance of DPPH distilled water solution, A_1_ represents the absorbance of hydrogels supernatant and DPPH methanol mixture, and A_2_ represents the absorbance of DPPH methanol solution.

### 2.9 Intracellular ROS scavenging activity

The accumulation of ROS was assessed using the radical probe DCFH-DA. Initially, RAW264.7 cells were seeded in a 24-well plate at a density of 1 × 10^5^ cells/mL and cultured for 24 h. The cells were then treated with hydrogel extracts. After 48 h of incubation, H_2_O_2_ (100 μM) was added, and the cells were incubated at 37 °C for an additional 2 h. The untreated group was labeled as the control group, while the group incubated with only H_2_O_2_ was designated as the positive group. After removing the culture medium containing H_2_O_2_, DCFH-DA diluted in PBS was added to the cells. The cells were incubated at 37 °C for 20 min in the dark. Following incubation, the cells were washed three times with PBS to remove any uninternalized DCFH-DA. Finally, the cells were visualized using an inverted fluorescence microscope.

### 2.10 Immunomodulatory effects of Lap-Gel/H-E-Cu hydrogels on RAW264.7 cells

For IF staining, macrophages were cultured in confocal Petri dishes at a density of 5 × 10^4^ cells/mL in 1 mL of medium. The following four groups were assessed for anti-inflammatory effects: (1) Control group: cells without treatment. (2) Negative control group: cells were treated with LPS, (100 ng/mL, Sigma-Aldrich, USA) or IL-4 (20 ng/mL, Peprotech, USA) for 48 h (3) Lap-Gel group: Negative control cells treated with Lap-Gel hydrogel extracts. (4) Lap-Gel/H-E-Cu group: Negative control treated with Lap-Gel/H-E-Cu hydrogels extracts. After 48 h of incubation, the macrophages were digested, centrifuged (1000 rpm, 5 min), then, cells were washed with PBS thrice and fixed with 4 % paraformaldehyde for 30 min at 4 °C. After 48 h of incubation, the macrophages were digested, centrifuged (1000 rpm, 5 min), washed three times with PBS, and fixed with 4 % paraformaldehyde for 30 min at 4 °C. The cells were then permeabilized with 0.5 % Triton X-100 for 30 min and incubated with 5 % goat serum for 1.5 h. Primary antibodies against iNOS (1:50, Proteintech, China) or CD206 (1:50, Proteintech, China) were applied and incubated at 4 °C for 12 h. Afterward, the cells were incubated with Alexa Fluor 647-conjugated secondary antibody (1:200, Cell Signaling Technology, USA) for 1 h in the dark. The cells were stained with DAPI for 30 min, and a drop of anti-fluorescence quencher was added to each dish. The red fluorescence intensity was observed using a confocal microscope (Leica, Germany), and ImageJ software was used to quantify the fluorescence intensity.

### 2.11 Osteogenic differentiation effects of Lap-Gel/H-E-Cu hydrogels on BMSCs

BMSCs (5 × 10^4^ cells/mL, 500 μL) were plated in 12-well plates and cultured for 24 h. The supernatant was then discarded and replaced with hydrogel extracts, followed by incubation for four days. ALP activity in BMSCs was evaluated using a BCIP/NBT staining kit (Beyotime, China). Additionally, after BMSCs were incubated in osteogenic induction medium for 21 days, calcium deposits were evaluated using an alizarin red staining kit (Solarbio, China) following the manufacturer's instructions. Images were captured using a scanner. For IF, BMSCs (5 × 10^4^ cells mL^−1^, 1 mL) were seeded in confocal Petri dishes and cultured for 7 days and stained for osteogenic protein RUNX2 and OCN. The cells were fixed 4 % paraformaldehyde for 30 min. BMSCs were treated with 0.5 % Triton X-100 for 30 min to permeabilize the cells, followed by blocking with 5 % goat serum for 1.5 h. They were then incubated overnight at 4 °C with Anti-RUNX2 (1:100, Abcam, USA) and Anti-OCN (1:100, Cell Signaling Technology, USA). Afterward, Alexa Fluor 647 Conjugate was applied for 1 h at room temperature in the dark. The cytoskeleton and nuclei were stained with rhodamine phalloidin (Thermo Fisher Scientific, Waltham, MA, USA) and DAPI for 30 min. Finally, an anti-fluorescence quencher was added, and fluorescence intensity was observed using a confocal microscope. ImageJ software was used for 3D surface mapping.

### 2.12 *In vivo* establishment of a rat periodontitis model

All animal experiments were conducted following the guidelines of the Animal Care Committee and were approved by the Ethics Committee of Southwest Medical University (Document No.: 20221214-005). Twenty male SD rats (6 weeks old, 180–220 g) underwent a one-week acclimation period before being randomly assigned to four groups: (1) Control group, receiving no treatment; (2) Periodontitis group, with periodontitis induced via ligation; (3) Lap-Gel group, where induced periodontitis was treated with Lap-Gel hydrogels; and (4) Lap-Gel/H-E-Cu group, where induced periodontitis was treated with Lap-Gel/H-E-Cu hydrogels.

Rats were anesthetized with isoflurane (2 % in 100 % oxygen), and a 0.2 mm orthodontic arch wire was securely inserted beneath the gingiva of the bilateral maxillary first molars to induce periodontitis. After two weeks of ligature-induced periodontitis, the arch wire was removed. And the severity of periodontal inflammation was evaluated to ensure consistency across all groups. Hydrogels (0.1 mL/kg) were then administered directly into the periodontal defect sites using a 5 mL syringe fitted with a clinical-grade needle (26G). To enhance therapeutic retention and effectiveness, hydrogel injections were performed five times at 3-day intervals.

### 2.13 Micro-CT analysis

After two weeks of hydrogel treatment, rats were euthanized with excess isoflurane. Maxillary bone samples were fixed in 4 % paraformaldehyde for 48 h, washed three times with PBS, and scanned using a nanoVoxel100 scanner (Tianjin, China) with a 3.8 μm resolution. 3D models and 2D images were reconstructed using Avizo software (Thermo Fisher, USA). The images were analyzed using ImageJ software to measure the distance between the cemento-enamel junction (CEJ) and the alveolar bone crest (ABC) in 2D. The inter-root regions of the distal buccal roots of the maxillary first molars and the proximal buccal roots of the maxillary second molars were designated as the regions of interest (ROI). The first slice of the CEJ served as the upper boundary of the ROI, while the lower boundary was defined by the slice extending down to the distal tip of the maxillary first molar that disappeared first.

### 2.14 Histology and immunohistochemistry

The maxillary bone samples were fixed in 4 % paraformaldehyde and decalcified in a 10 % ethylenediaminetetraacetic acid (EDTA) solution for 30 days. After decalcification, the samples were embedded in paraffin and sectioned into 4-μm thick slices. These slides were stained with H&E and Masson's trichrome and scanned using a digital pathology slide scanner (KF-PRO-002, China). Immunohistochemical staining was also performed to evaluate the expression of osteocalcin (OCN), bone morphogenetic protein-2 (BMP-2), and inflammatory cytokines (IL-10 and TNF-α).

### 2.15 Statistical analysis

Statistical analysis was performed using one-way analysis of variance (ANOVA) and independent t-tests. Data are expressed as mean ± standard deviation (SD) with n ≥ 3. Statistical significance was set as follows: ∗*P* < 0.05, ∗*P* < 0.01, ∗∗∗*P* < 0.001, and ∗∗∗∗*P* < 0.0001.

## 3 Results and discussions

### 3.1 Characterization of EGCG-Cu coordination compound

In this study, a metal-polyphenol coordination network was constructed using EGCG and Cu^2+^. EGCG possesses antioxidant properties, effectively scavenging excessive ROS. However, its low bioavailability and poor stability as a small molecule limit its biomedical applications. To overcome these limitations, copper ions were introduced to form a metal-phenol coordination complex, which enhances the stability of the composite *in vivo* and extends its retention time, thereby improving its therapeutic efficacy.

**Fig. 2:**
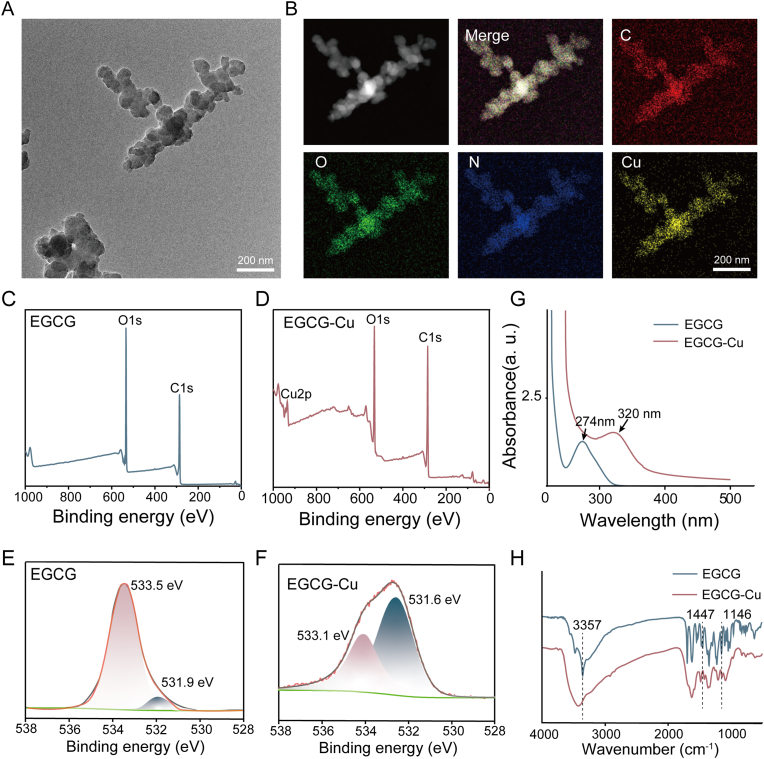
Morphological and elemental characterization of EGCG-Cu. (A) TEM images of EGCG-Cu. (B) The elemental mapping images of EGCG-Cu complexing compound. (C) XPS spectrogram of EGCG. (D) XPS spectrogram of EGCG-Cu. (E) O1s XPS spectra of EGCG. (F) O1s XPS spectra of EGCG-Cu. (G) UV–vis absorption spectra of EGCG and EGCG-Cu. (H) FTIR spectra of EGCG and EGCG-Cu.

As shown in Fig. 2A, TEM revealed the morphology of EGCG-Cu complexes, confirming their successful synthesis as nanoparticles. The elemental mapping of the EGCG-Cu complex (Fig. 2B) shows a consistent distribution of carbon (C), oxygen (O), and copper (Cu) elements, confirming the effective coordination of Cu^2+^ with EGCG and the successful synthesis of the EGCG-Cu composite material. Fig. 2C and D presents the XPS spectra of EGCG and EGCG-Cu, where two peaks corresponding to the C1s and O1s states are observed for EGCG [21], while an additional Cu2p peak appears for the EGCG-Cu complex [31]. These results confirm the presence of Cu within the complex, indicating the successful synthesis of EGCG-Cu.

Fig. 2E and F shows the O1s XPS spectra for EGCG and EGCG-Cu, respectively. The results distinctly show that EGCG features a primary peak at 533.5 eV, attributed to the HO-C group, along with a secondary peak at 531.9 eV, corresponding to the C=O group [32]. Following coordination with Cu^2+^, the O1s peak corresponding to the HO-C group shifts from 533.5 eV to 533.1 eV, indicating a reduction in binding energy, whereas the O1s peak related to the C=O group moves from 531.9 eV to 531.6 eV, reflecting an increase in binding energy. This shift suggests electron transfer from Cu^2+^ to EGCG, with all findings aligning with previous studies [33,34].

The EGCG-Cu complex was characterized using UV–Vis spectroscopy. As shown in Fig. 2G, a characteristic peak of EGCG appears at 274 nm. However, the UV–Vis spectrum of the EGCG-Cu reaction solution displays a notable shift, with the peak moving from 274 nm to 320 nm. This redshift of 46 nm in the absorbance wavelength confirms that Cu^2+^ interacts with EGCG, altering its spectral properties [35]. The complexation between EGCG and Cu^2+^ in solution leads to the formation of a complex with a specific stereo structure, which accounts for this observed redshift [21,33,35]. In addition, FTIR spectroscopy was utilized to analyze the EGCG and EGCG-Cu solutions throughout the preparation process. The distinctive absorption bands of EGCG are observed at 3457 cm^−1^, 1447 cm^−1^, and 1146 cm^−1^. Following complexation with Cu^2+^, the phenolic hydroxyl group's vibration peak (originally at 3356 cm^−1^) shifts to a higher wavenumber (3425 cm^−1^), possibly due to partial hydrogen bond disruption within EGCG, indicating a complexation reaction between Cu^2+^ and EGCG (Fig. 2H) [21]. Additionally, in the Cu-EGCG complex, the deformation vibration of the aromatic ring (1447 cm^−1^) and the stretching vibration of the C-O-H group (1146 cm^−1^) are weaker than in EGCG. These findings suggest that Cu^2+^ interacts with the phenolic hydroxyl groups of EGCG, replacing the hydrogen in the O-H bond and resulting in the formation of an O-Cu bond. Thus, EGCG-Cu complex was successfully prepared, according to the FTIR, XPS, and UV–Vis spectrum results.

### 3.2 Characterization of the Lap-Gel/H-E-Cu hydrogels

**Fig. 3:**
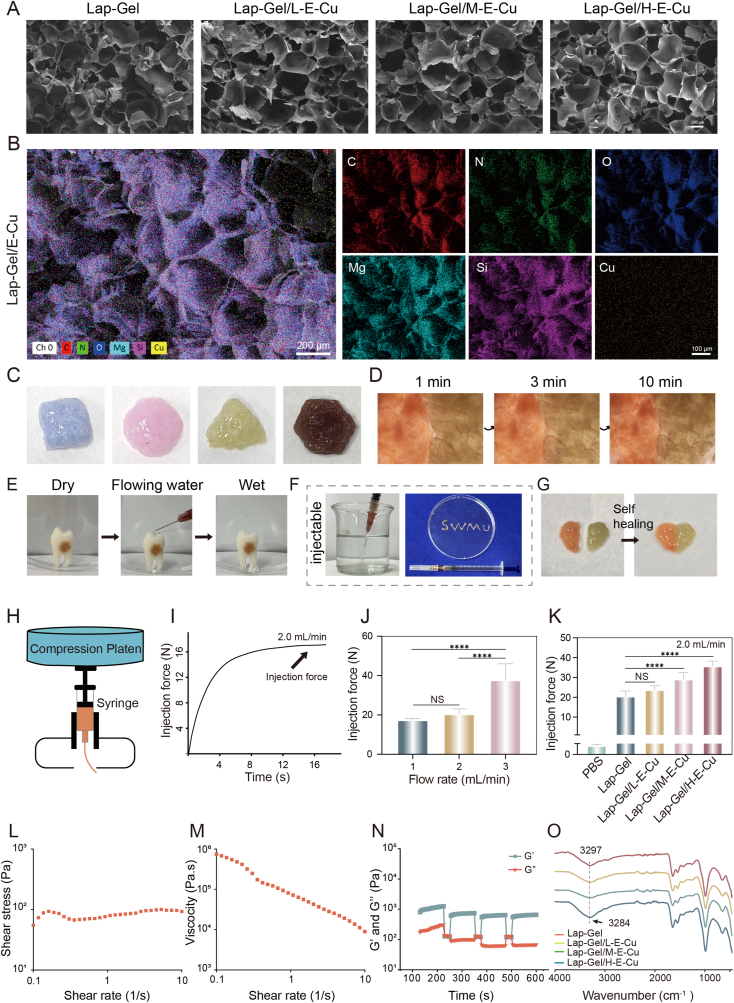
Characterization of the Lap-Gel/H-E-Cu hydrogels. (A) The SEM images of the Lap-Gel, Lap-Gel/L-E-Cu, Lap-Gel/M-E-Cu, and Lap-Gel/H-E-Cu hydrogels. (B) Elemental mapping of Lap-Gel/H-E-Cu hydrogel. (C) Different shapes of hydrogels. (D) and (G) self-healing property of hydrogels over time (E) Adhesion to premolar. (F) Injectability of hydrogels through the syringe. (H) Schematic representation of testing hydrogel injection force with mechanical tester. (I) Extrusion force vs time was recorded, and the arrow indicates the maximum required force to injecting the hydrogel. (J) Injection force of Lap-Gel was determined in different velocities of flow. (K) Injection force of Lap-Gel, Lap-Gel/L-E-Cu, Lap-Gel/M-E-Cu, and Lap-Gel/H-E-Cu. (L) Shear stress versus shear rate for the Lap-Gel/H-E-Cu hydrogels. (M) Viscosity of the Lap-Gel/H-E-Cu hydrogels as a function of shear rate. (N) The G′ and G″ values were recorded when the Lap-Gel/H-E-Cu hydrogels were subjected to cyclic strain changes between 0.1 % and 100 % strain. (O) FTIR spectra of Lap-Gel, Lap-Gel/L-E-Cu, Lap-Gel/M-E-Cu, and Lap-Gel/H-E-Cu hydrogels.

To ensure the precise delivery of EGCG-Cu to the periodontal pocket, a hydrogel was employed as a drug delivery scaffold. With its excellent fluidity and injectability, the hydrogel offers high clinical applicability, making it an ideal carrier for localized periodontal treatment. In this study, Gelatin combined with synthetic Laponite was used to prepare the injectable hydrogel scaffold. Fig. 3A shows the SEM image of the hydrogel's cross-sectional morphology, exhibiting an interconnected 3D porous network structure. This structure creates favorable channels for cell growth, migration, and metabolism, thereby promoting bone defect repair [36]. Moreover, the EDS mapping images (Fig. 3B) confirm the uniform distribution of C, N, O, Mg, Si, and Cu within the Lap-Gel/H-E-Cu hydrogel. Notably, the Cu content reaches 1.19 %, demonstrating the successful integration of EGCG-Cu into the hydrogel matrix.

FTIR spectra were used to detect the functional groups in Lap-Gel, Lap-Gel/L-E-Cu, Lap-Gel/M-E-Cu, and Lap-Gel/H-E-Cu hydrogels (Fig. 3O). No significant changes in the waveforms of the hydrogels were observed, confirming that the addition of EGCG-Cu did not disrupt the chemical structure of the Lap-Gel hydrogel. The characteristic peak of the Lap-Gel hydrogel appears at 3297 cm^−1^, corresponding to the amide-A band in the gelatin structure [37]. Compared to Lap-Gel, the amide-A band in Lap-Gel/H-E-Cu shifted to a lower wavenumber with the addition of EGCG-Cu, from 3297 cm^−1^ to 3284 cm^−1^. This shift could result from the hydrogen bond formation between the N-H groups in gelatin molecules and the -OH groups in EGCG [38]. As the degree of hydrogen bonding between N-H and EGCG increases, the absorption peak shifts to a lower frequency. These results suggest that EGCG can interact with gelatin molecules, forming a more compact network structure.

To evaluate the release kinetics of the active components, the cumulative release of Cu^2+^ and EGCG from the Laponite/gelatin hydrogel was assessed over 90 h (Figure S1 and Figure S2). Cu^2+^ exhibited a biphasic release profile, characterized by an initial burst release within the first 20 h, followed by a slower, sustained release phase, ultimately reaching approximately 33.3 % cumulative release at 90 h. In contrast, EGCG showed a more controlled and gradual release, with a cumulative release of ∼18.95 % at 90 h. The slower release of EGCG compared to Cu^2+^ is likely due to the strong coordination interaction between EGCG and Cu^2+^, which stabilizes the complex and hinders the immediate dissociation of EGCG. Additionally, hydrogen bonding and electrostatic interactions between EGCG and the Laponite/gelatin matrix may further restrict its diffusion within the hydrogel. These physicochemical interactions collectively contribute to the more sustained release of EGCG. The differential release behavior of Cu^2+^ and EGCG suggests a potentially advantageous sequential delivery mechanism. The initial burst release of Cu^2+^ may facilitate rapid antibacterial action, while the prolonged release of EGCG can provide lasting antioxidant and immunomodulatory effects, thus supporting both infection control and tissue regeneration in the periodontal environment.

In addition, the *in vitro* degradation results (Fig. S5) showed a gradual mass loss of the hydrogel in PBS at 37 °C over a 7-day period. Specifically, the hydrogel retained 74.81 % of its initial mass on day 1, 61.14 % on day 3, 49.64 % on day 5, and 31.87 % on day 7. These results indicate a moderate and continuous degradation trend. The fact that over 60 % of the hydrogel mass remains within the first 3 days suggests that the material maintains sufficient structural integrity to support therapeutic effects (e.g., drug release, space maintenance) during the initial post-application period. Furthermore, the gradual degradation may facilitate natural tissue remodeling and reduce long-term foreign material burden.

Subsequently, to enable the observation of the hydrogel's plasticity and self-healing properties, it was dyed in various colors using colored solutions. It was observed that the hydrogel could be molded into various shapes, such as squares, circles, triangles, and hexagons (Fig. 3C). Additionally, two separate hydrogel pieces could self-heal into a single piece within 10 min of contact (Fig. 3G), with the contact interface gradually becoming indistinguishable under magnification (Fig. 3D). These properties indicate that when applied to periodontal pockets, the hydrogel can adapt well to irregular bone defects and form a physical barrier to prevent bacterial invasion. Given that periodontal tissue is a wet environment subjected to gingival crevicular fluid flow [39], the hydrogel must possess excellent wet adhesion properties to ensure prolonged drug release at the target site. As shown in Fig. 3E and Supplementary Video S1, the Lap-Gel/H-E-Cu hydrogel demonstrated strong adhesion to moist tooth root surfaces and retained its position even under continuous water flow. This robust retention is primarily attributed to multiple interactions—including hydrogen bonding, electrostatic attraction, and hydrophobic effects—which collectively contribute to prolonged drug residence at the site of periodontal lesions [40].

To further assess retention under physiologically relevant conditions, we applied the hydrogel onto excised maxillary periodontal tissue from rats. As shown in Fig. S4, the hydrogel adhered firmly to the tissue surface in both dry and wet states, the latter simulated by phosphate-buffered saline (PBS) immersion. Notably, even after prolonged PBS exposure, the hydrogel maintained close contact with the tissue, indicating excellent adhesion and environmental stability. These findings support the hydrogel's ability to remain localized within the periodontal pocket and deliver sustained therapeutic effects in the challenging oral environment. Furthermore, the hydrogel's shear-thinning and self-healing properties, imparted by the Laponite/gelatin network, allow it to adapt to the irregular contours of oral tissues and maintain stability in moisture-rich environments. These characteristics collectively enhance its potential for sustained local therapy in periodontal applications.

In addition, manual injection tests were conducted to demonstrate the practical injectability of the hydrogel under clinically relevant conditions. As shown in Fig. 3F, the hydrogel could be extruded into strips through a syringe. To replicate the injection process, the injectability of the hydrogel was evaluated using clinical needles. A universal mechanical testing machine was utilized to measure the extrusion force needed to inject the hydrogel from a 5 mL syringe at a predetermined flow rate. As shown in Fig. 3H, the impact of various injection speeds and hydrogel formulations on the injection force was assessed. The extrusion force of Lap-Gel hydrogel during mechanical compression (2 mL/min) was measured (Fig. 3I), and the maximum injection force of the hydrogel was recorded at 17.5 N. When injecting Lap-Gel hydrogel at flow rates of 1 mL/min, 2 mL/min, and 3 mL/min, it was found that the average injection force increased with flow rate, reaching a maximum of 37.60 ± 8.44 N at 3 mL/min (Fig. 3J). When the injection speed was set at 2 mL/min, the required injection force increased with the EGCG-Cu loading, with the Lap-Gel/H-E-Cu group exhibiting the highest injection force (35.59 ± 2.69 N) (Fig. 3K). Studies have shown that polyphenols can directly physically interact with polymers and stabilize scaffold structures through non-covalent interactions [41]. Therefore, the increased injection force of the Lap-Gel/H-E-Cu hydrogel may result from non-covalent reactions between EGCG and gelatin [40,41], which enhance the hydrogel's structural stability and reduce its shear-thinning ability, making the sol-gel transition more difficult during injection.

To further assess the injectability under conditions closer to clinical practice, we repeated the injection force measurements using a 5 mL syringe fitted with a 22G needle (Fig. S3). The maximum injection force in this setup was reduced to approximately 16 N, suggesting improved practicality and ease of application in clinical settings. It is worth noting that earlier measurements were performed using a 5 mL syringe with a 26G needle, which was selected for compatibility with small animal experiments. However, smaller-diameter needles and lower syringe volumes typically lead to increased injection resistance during mechanical testing. In actual clinical procedures, the use of larger syringe and needle sizes, such as 5 mL syringes and 22G or 24G needles, is more common and facilitates smoother administration. Additionally, a supplementary demonstration video (Video S2) has been provided to visually illustrate that the hydrogel can be manually injected into PBS solution, further supporting its clinical operability.

Furthermore, rheological tests were performed to investigate the shear-thinning behavior of the prepared hydrogel. The relationships between shear stress, viscosity, and shear rate were studied (Fig. 3L and M). The results indicated that at low shear rates, the shear stress rapidly increased to a stable level before decreasing to negligible levels at high shear rates. This suggests that the hydrogel injection is controllable. Furthermore, the viscosity of the hydrogel decreases as shear rates increase (Fig. 3M), demonstrating shear-thinning behavior resulting from the disruption of reversible interactions within the hydrogel network. The storage modulus (G′) and loss modulus (G″) of the hydrogel were assessed at 37 °C using the ElastoSens™ Bio2, a non-destructive technique for evaluating the mechanical properties of viscoelastic materials. Fig. 3N illustrates the recovery behavior after repeated applications of high and low strain. When subjected to high-strain shear rates, the storage modulus significantly decreased, and the hydrogel exhibited liquid-like behavior. In contrast, at low-strain shear rates, the storage modulus markedly increased, with the hydrogel displaying solid-like behavior. These results demonstrate that after exposure to high-strain shear rates, the hydrogel successfully recovers to a solid-like state, suggesting its ability to restore its semi-solid or solid properties quickly after injection. Additionally, multiple cycling tests further demonstrated that the hydrogel could continuously return to its original modulus level after several shear damage cycles, showcasing excellent self-healing abilities. This self-healing performance is crucial for maintaining the structural integrity of the material during the injection molding process and helps prevent uncontrolled flow of the material during injection [42].

### 3.3 Antibacterial properties Lap-Gel/H-E-Cu hydrogels *in vitro*

**Fig. 4:**
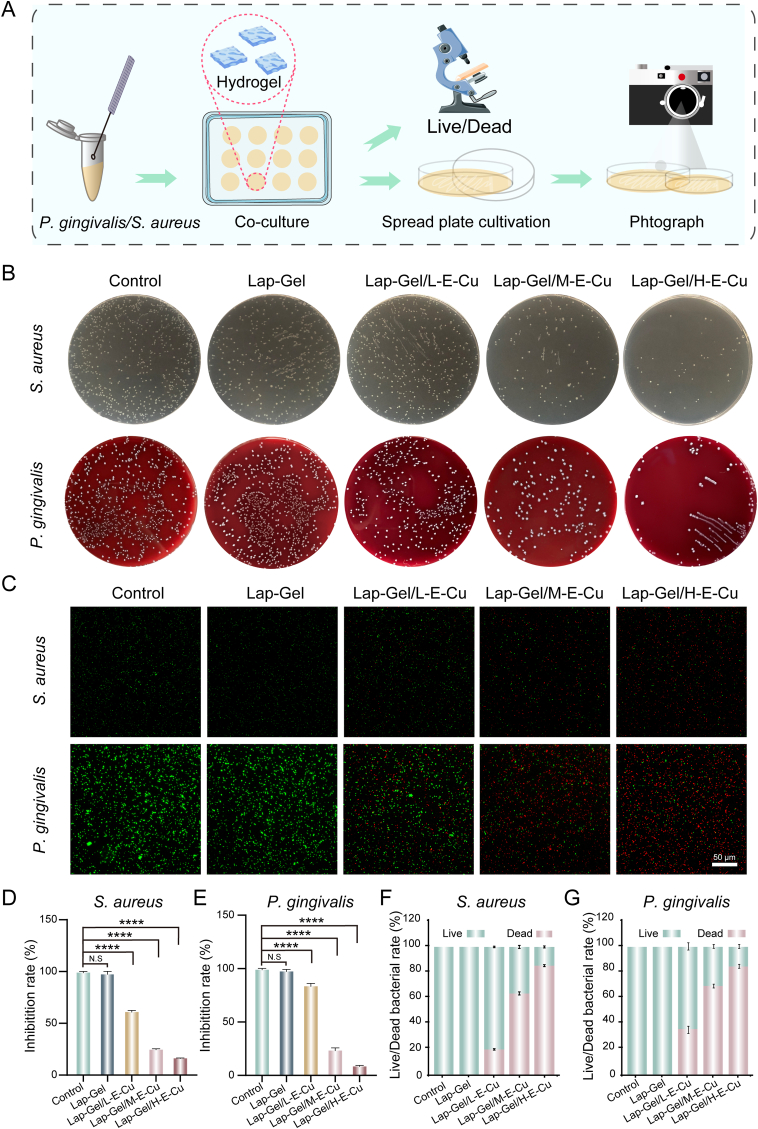
*In vitro* antibacterial properties of the hydrogels. (A) Schematic diagram of antibacterial experiment steps. (B) Images of *S. aureus* and *P. gingivalis* bacterial colonies treated with different hydrogel groups. (C) Live/dead vitality image of *S. aureus* and *P. gingivalis* treated with various hydrogels. (D) Quantitative analysis of the antimicrobial rates against *S. aureus.* (E) Quantitative analysis of the antimicrobial rates against *P. gingivalis*. (F) Live/dead bacterial rate of *S. aureus* treated with various hydrogels. (G) Live/dead bacterial rate of *P. gingivalis* treated with various hydrogels.

Dental plaque is a trigger for periodontitis, and *P. gingivalis* has been identified as a critical factor in disease progression [43]. *P. gingivalis* releases multiple virulence factors that induce inflammation, leading to tissue destruction [3,7]. Given the unique role of bacteria in periodontitis, biomaterials intended to treat this disease must possess strong antibacterial properties [44]. To evaluate the antibacterial effect of the composite hydrogel on *P. gingivalis*, we incubated the hydrogel with *P. gingivalis* and *S. aureus* (Fig. 4A). The data shown in Fig. 4B indicate that, compared with the control group, there was no significant difference in colony counts on blood agar plates in the Lap-Gel hydrogel-treated group. In contrast, colony counts on blood agar plates decreased in the Lap-Gel/L-E-Cu, Lap-Gel/M-E-Cu, and Lap-Gel/H-E-Cu hydrogel-treated groups. Furthermore, compared with the Lap-Gel group, the reduction in colony counts was more pronounced in the Lap-Gel/H-E-Cu group than in the Lap-Gel/L-E-Cu and Lap-Gel/M-E-Cu groups.

To further evaluate the antibacterial effect of the composite hydrogel, live and dead bacteria were stained with fluorescence. Live bacteria showed green fluorescence, while those with damaged cell membranes displayed red fluorescence. The bacterial suspensions treated with each hydrogel group were then collected and stained. As presented in Fig. 4C, the control and Lap-Gel groups exhibited primarily green fluorescence, indicating that the hydrogel scaffold lacks significant antibacterial properties. In contrast, the Lap-Gel/L-E-Cu, Lap-Gel/M-E-Cu, and Lap-Gel/H-E-Cu hydrogel-treated groups showed areas of red fluorescence, indicating bacterial death in these groups. Fluorescence analysis results (as shown in Fig. 4F and G) demonstrated varying inhibition rates against *P. gingivalis* and *S. aureus* in the Lap-Gel/L-E-Cu, Lap-Gel/M-E-Cu, and Lap-Gel/H-E-Cu groups, with the Lap-Gel/H-E-Cu group showing the strongest inhibitory effect. Collectively, the Lap-Gel/H-E-Cu hydrogel exhibited the strongest antibacterial performance, likely due to the synergistic release of copper ions and EGCG. Copper ions possess notable antibacterial properties, primarily through mechanisms such as disrupting cell membranes, DNA, and proteins [2,45,46]. EGCG, a natural tea polyphenol, has been shown to exert antibacterial effects by inhibiting protease activity, hemolytic activity, coagulation activity, biofilm formation, aggregation, and virulence gene expression in bacteria [33]. By incorporating varying concentrations of the EGCG-Cu complex, the hydrogel achieves different levels of antibacterial effectiveness.

### 3.4 Biocompatibility assay *in vitro*

**Fig. 5:**
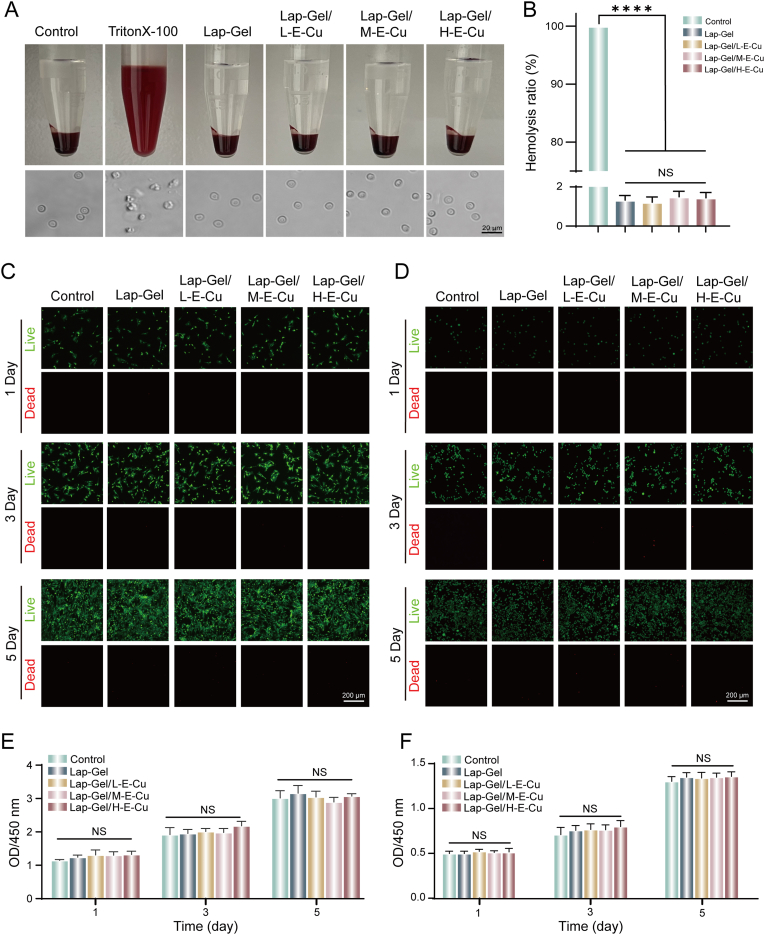
Hemocompatibility and cytocompatibility testing of hydrogels. (A) Blood image after co-culture with the hydrogel and erythrocyte morphology under the microscope. (B) Hemolysis rate of the hydrogels. Live/dead fluorescent staining of (C) RAW264.7 cells and (D) BMSCs. Cell viability of (E) RAW264.7 cells and (F) BMSCs at days 1, 3, and 5.

As a promising injectable biomaterial, this composite hydrogel must demonstrate excellent biocompatibility [47]. To assess the hemocompatibility of the composite hydrogel, we incubated it with fresh blood. Poor hemocompatibility in materials leads to acute erythrocyte destruction, causing hemolysis. Each hydrogel treatment group exhibited a clear supernatant, indicating the absence of significant hemolysis. Additionally, microscopic observation showed normal erythrocyte morphology in all groups (Fig. 5A and B). To further assess the cellular compatibility of the hydrogel, RAW264.7 and BMSCs were incubated with hydrogel extracts for 1, 3, and 5 days, followed by live/dead cell staining and CCK-8 assays. As shown in Fig. 5C and D, green fluorescence indicating live cells was predominantly observed on days 1, 3, and 5, while red fluorescence indicating dead cells was rarely observed, suggesting the Lap-Gel/H-E-Cu hydrogel has good cellular compatibility. The CCK-8 assay showed no significant difference in the viability of BMSCs treated with hydrogel extracts compared to the control group (Fig. 5E). Similarly, the CCK-8 assay for RAW264.7 cells revealed no noticeable toxicity (Fig. 5F). These results suggest that the hydrogel exhibits good biocompatibility. Based on the above experimental results, we found that the Lap-Gel/H-E-Cu hydrogel exhibited excellent biocompatibility and superior antibacterial performance compared to other hydrogel groups. Therefore, in subsequent experiments, we selected Lap-Gel/H-E-Cu hydrogel as our research focus and further validated its functions in ROS scavenging, immunomodulation, and promoting periodontal bone defect repair.

### 3.5 Multiple ROS scavenging capacities of Lap-Gel/H-E-Cu hydrogels

**Fig. 6:**
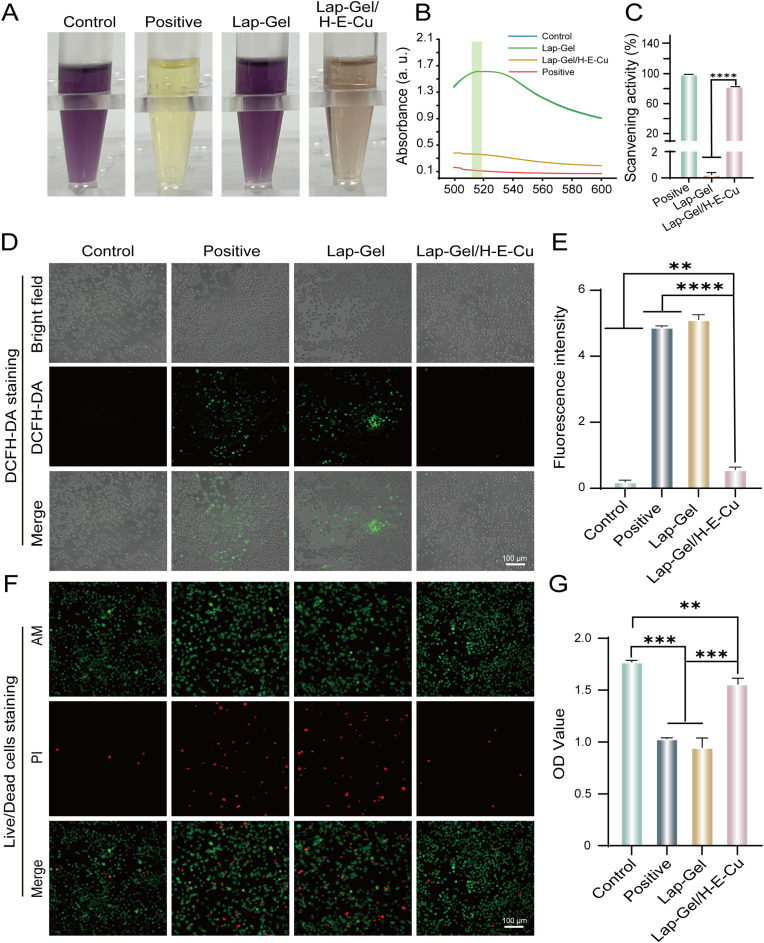
Antioxidant properties of hydrogels. (A) Absorbance curves changes of staining appearance in different groups of hydrogel extractions added to DPPH solution. Absorbance curves (B) and scavenging ratios (C) of hydrogels with varying concentrations of nanosheets of DPPH assays. (D) DCFH-DA staining and (E) fluorescent quantification results. (F) Live/dead staining and (G) CCK-8 assay under oxidative stress conditions.

Excessive ROS disrupts macrophage differentiation and promotes M1 macrophage polarization, resulting in an imbalance in the M1/M2 ratio [7,10,48]. In the pathological environment of periodontitis, this trend leads to oxidative stress damage in BMSCs, progressively contributing to the degradation of periodontal tissue [25]. Therefore, scavenging ROS presents a promising approach for the regeneration of periodontal tissue. In this study, the *in vitro* radical scavenging ability of Lap-Gel/E-Cu hydrogel was verified using the DPPH radical scavenging ability assay kit. DPPH is a stable organic free radical with a characteristic purple color [48]. When antioxidants interact with DPPH, they neutralize the free radicals, resulting in a color change from purple to yellow [48,49]. The antioxidant's free radical scavenging capacity can be quantified by measuring the reduction in absorbance at 515 nm [49]. As shown in Fig. 6A, after treatment with Lap-Gel/H-E-Cu hydrogel, the solution color faded from purple to yellow, and the absorbance at 515 nm significantly decreased (Fig. 6B). The DPPH scavenging rate of the hydrogel reached (83.4 ± 0.03 %) (Fig. 6C). These experimental results indicate that Lap-Gel/H-E-Cu hydrogel exhibits excellent free radical scavenging potential.

To further demonstrate the ROS-scavenging properties of Lap-Gel/H-E-Cu, 2′,7′-dichlorodihydrofluorescein diacetate (DCFH-DA) staining was used to assess the total free radical levels in RAW264.7 cells after hydrogel incubation. DCFH-DA undergoes enzymatic hydrolysis to form DCFH, which is then oxidized by H_2_O_2_ to generate highly fluorescent DCF [15,49]. As shown in Fig. 6D, the fluorescence signal in the H_2_O_2_ group was stronger than in the control group. The fluorescence intensity in the Lap-Gel-treated group showed no significant difference from that of the H_2_O_2_ group, suggesting it lacked ROS-scavenging ability. In contrast, the Lap-Gel/H-E-Cu-treated group exhibited lower fluorescence intensity than the H_2_O_2_ group, demonstrating a strong ROS-scavenging capability (Fig. 6E). This effect stems from the polyphenol groups in EGCG, which efficiently neutralize excess ROS [33].

Due to the potent ROS-scavenging ability of Lap-Gel/H-E-Cu, its capacity to alleviate oxidative stress in RAW264.7 cells was examined *in vitro* through the CCK-8 assay and live/dead cell staining. As shown in Fig. 6F, a large number of dead cells (red fluorescence) were observed in the H_2_O_2_ group. In contrast, the Lap-Gel/H-E-Cu group exhibited significantly fewer dead cells. Additionally, the CCK-8 results showed a notable increase in cell viability in the Lap-Gel/H-E-Cu group compared to the H_2_O_2_ group (Fig. 6G). This finding suggests that Lap-Gel/H-E-Cu can restore cell viability compromised by excess ROS. These results confirm that Lap-Gel/H-E-Cu hydrogel effectively protects cells from oxidative stress by scavenging excess ROS, indicating its potential for treating periodontitis.

### 3.6 Cellular immunomodulation assay

Macrophages play a crucial role in the human body [50], with major functions including immune defense, immune regulation, immune secretion, tissue regeneration, and repair. Moreover, macrophages exhibit high plasticity, allowing them to be activated into different phenotypes under the magnetic stimulation of various environmental signals, performing distinct functions [7,51], Based on activation type, they are classified into the pro-inflammatory phenotype (M1) and the anti-inflammatory phenotype (M2) [10]. M1 macrophages are activated in response to bacterial infection, pathogenic molecules, or inflammatory factors (such as IFN-γ, LPS), producing large amounts of pro-inflammatory cytokines (e.g., TNF-α, IL-1β, IL-6, IL-12) and ROS, thus enhancing local inflammatory responses [10,48]. In the acute stage or early phase of chronic inflammation in periodontitis, M1 macrophages promote the inflammatory response and cytokine secretion, aiding in infection clearance [52]. In contrast, M2 macrophages are activated by signals like IL-4, IL-13, and TGF-β, and perform anti-inflammatory, tissue repair, and immunosuppressive functions. They secrete anti-inflammatory cytokines and support tissue repair and regeneration [7,48]. During the chronic stage of periodontitis or in the recovery phase after treatment, M2 macrophages play a critical role in repair. By secreting anti-inflammatory cytokines (such as IL-10 and TGF-β) and enhancing osteoblast function, M2 macrophages help suppress excessive inflammatory responses and contribute to alveolar bone reconstruction and periodontal tissue repair [53,54]. Imbalance in macrophage polarization may be a significant factor in the progression of periodontitis [55]; therefore, modulating macrophage polarization could provide new strategies for periodontitis treatment.

**Fig. 7:**
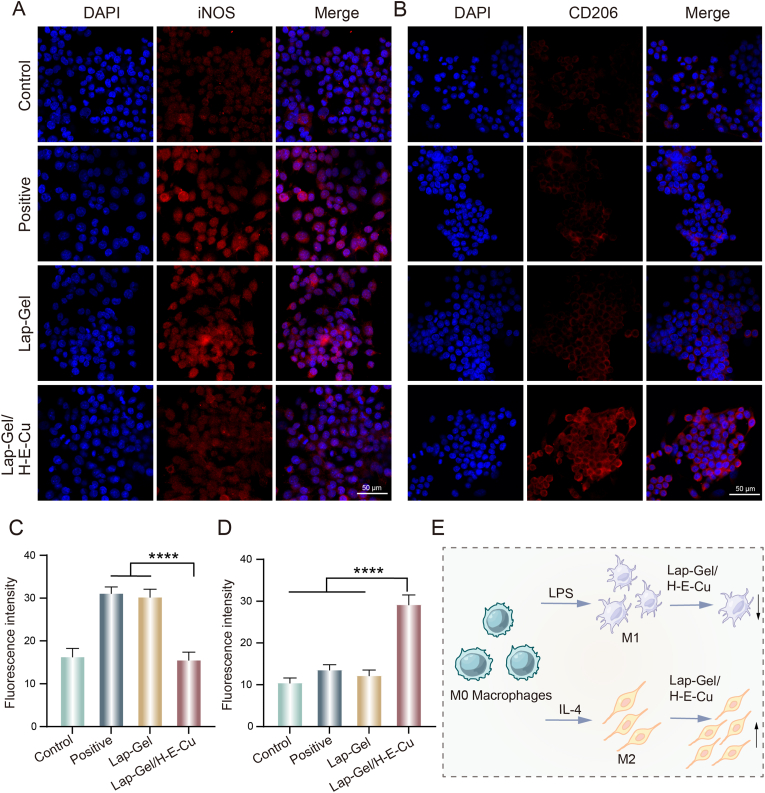
Macrophage polarization modulation by hydrogels. IF images showing hydrogels modulating macrophage polarization for (A) M1-type macrophage markers (iNOS) and (B) M2-type macrophages markers (CD206). (C) Fluorescence intensity of iNOS. (D) Fluorescence intensity of CD206. (E) Schematic diagram showing the effect of hydrogels on macrophage polarization.

In this study, LPS and IL-4 were used to induce macrophages to polarize into M1 and M2 phenotypes, respectively, to evaluate the immunomodulatory effect of Lap-Gel/H-E-Cu hydrogel on macrophage phenotypes. iNOS and CD206 were chosen as markers for M1 and M2 macrophages, respectively, for fluorescent labeling. As shown in Fig. 7A and C, compared to the positive control group (drug-induced polarization), the fluorescence intensity of iNOS in the Lap-Gel/H-E-Cu hydrogel group was significantly reduced. Conversely, compared to the positive control, the CD206 fluorescence intensity in the Lap-Gel/H-E-Cu hydrogel group was significantly higher than that in the positive control (Fig. 7B and D). The immunofluorescence results indicate that Lap-Gel/H-E-Cu hydrogel can regulate macrophage polarization, demonstrating strong anti-inflammatory, immunoregulatory, and antioxidant potential by suppressing M1 polarization and enhancing M2 polarization. (Fig. 7E).

### 3.7 Osteogenic differentiation effects of Lap-Gel/H-E-Cu hydrogels on BMSC

**Fig. 8:**
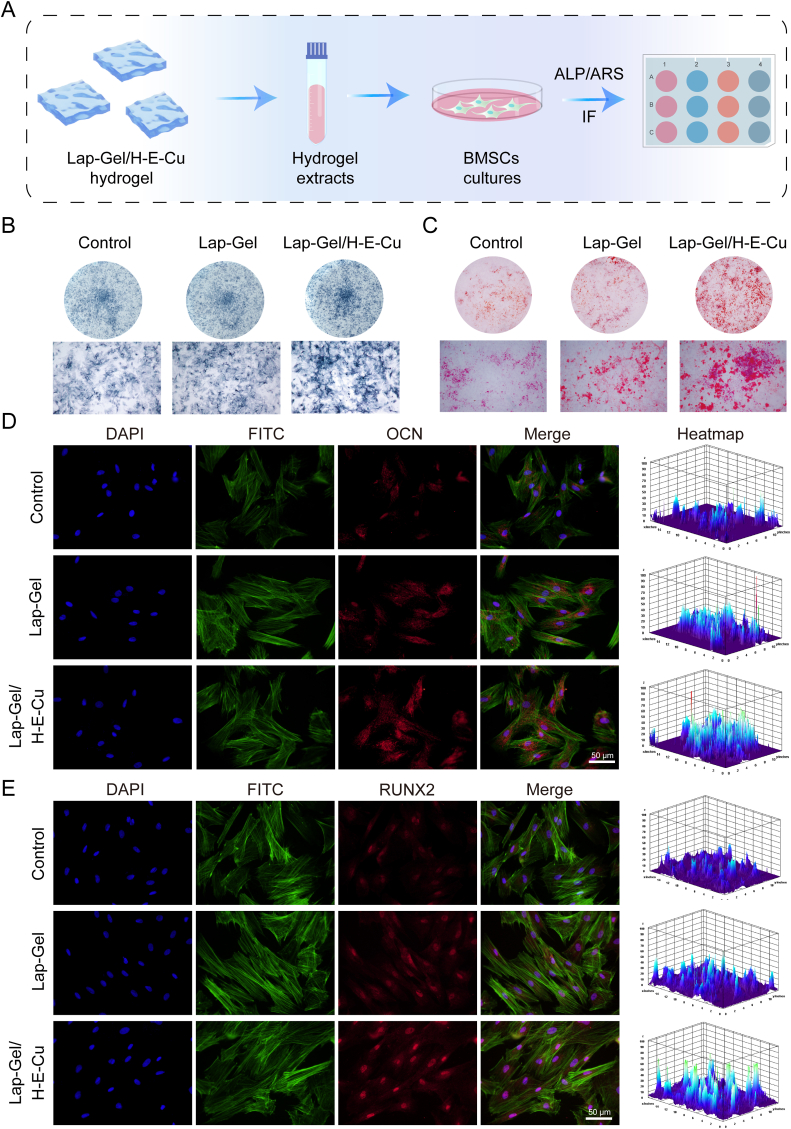
*In vitro* assessment of the osteogenic differentiation potential of hydrogels. (A) Schematic diagram outlining the experimental procedure for ALP, ARS, and IF staining to evaluate BMSC osteogenic differentiation. (B) ALP staining (day 4) and ARS (day 21) of BMSCs respectively. (D) IF staining images of OCN. (E) IF staining images of RUNX2.

As an adult stem cell derived from the mesoderm, BMSCs exhibit remarkable self-renewal and multi-lineage differentiation potential, enabling differentiation into various mesenchymal tissues [56]. The inflammation caused by periodontitis results in alveolar bone resorption and destruction, where alveolar bone loss is the primary cause of tooth mobility and eventual loss [57]. Promoting the differentiation of BMSCs into osteoblasts can accelerate new bone formation [58], filling the bone defects created by periodontitis, thereby reconstructing the alveolar bone structure and anchoring teeth securely [59]. Studies have demonstrated that BMSCs, under specific osteoinductive conditions, can form new bone tissue analogous to native bone, thus functionally supporting the dentition [60,61]. To assess the osteogenic effects of Lap-Gel/H-E-Cu hydrogel on BMSCs, this study conducted multiple evaluations to validate and analyze the osteoinductive properties of Lap-Gel/H-E-Cu hydrogel (Fig. 8A). ALP, as a phenotypic marker of osteogenic cells, directly reflects osteogenic activity and function, serving as a critical indicator for assessing cellular bone mineralization potential [62]. As illustrated in Fig. 8B, ALP staining was observed in all three groups. On day 4, compared to the control group, both the Lap-Gel group and the Lap-Gel/H-E-Cu group exhibited higher ALP activity, with ALP expression being highest in the Lap-Gel/H-E-Cu group. Additionally, in the ARS staining results, both the Lap-Gel and Lap-Gel/H-E-Cu groups showed a greater number of mineralized nodules than the control group, with the Lap-Gel/H-E-Cu group exhibiting an enhanced capacity for mineralized nodule formation (Fig. 8C).

In the process of bone tissue reconstruction, numerous cytokines play essential roles [63]. Among them, osteocalcin (OCN), a non-collagenous bone matrix protein synthesized by osteocytes, is critical in late-stage bone metabolism [28,64], while RUNX2 governs the osteogenic differentiation of mesenchymal stem cells and serves as a key regulatory factor in bone formation [64,65]. To further investigate the regulatory effects of Lap-Gel/H-E-Cu hydrogel on BMSC osteogenic differentiation, immunofluorescence staining was employed. As shown in Fig. 8D and E, the protein expression levels of osteogenic markers OCN and RUNX2 were significantly upregulated in cells treated with both Lap-Gel and Lap-Gel/H-E-Cu compared to the Control group, with the highest expression levels observed in the Lap-Gel/H-E-Cu group. These findings indicate that the Lap-Gel hydrogel scaffold itself possesses intrinsic osteoinductive properties, while the incorporation of the EGCG-Cu complex further enhances osteogenesis by synergistically promoting the expression of osteogenesis-related factors. The enhanced osteoinductive properties observed in the Lap-Gel/H-E-Cu hydrogel may be attributed to the sustained release of bioactive Cu^2+^ ions, which upregulate key osteogenic genes such as Runx2, ALP, and OCN, and stimulate angiogenic factors like VEGF [33]. Additionally, copper ions have been shown to activate intracellular signaling pathways, including PI3K/Akt and MAPK, thereby promoting the proliferation and osteogenic differentiation of mesenchymal stem cells. Together, these synergistic mechanisms likely account for the superior osteogenic performance of the Lap-Gel/H-E-Cu hydrogel observed in this study.

### 3.8 *In vivo* bone regeneration evaluation of Lap-Gel/H-E-Cu hydrogels

**Fig. 9:**
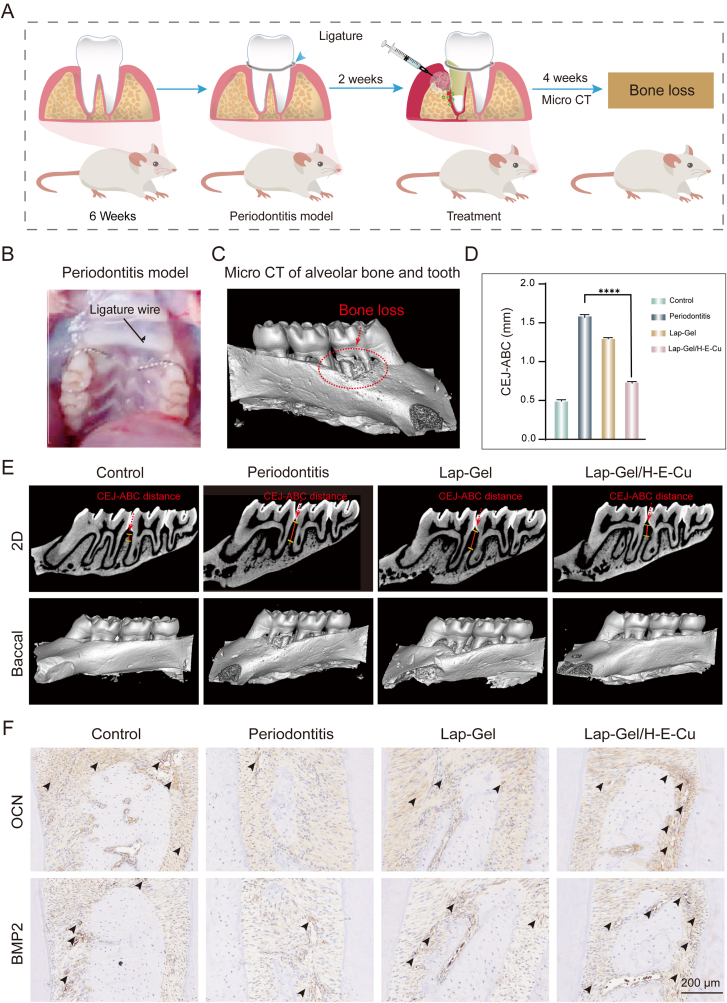
Bone regeneration assessments *in vivo*. (A) The schematic diagram of hydrogel treatment of periodontitis in SD rats. (B) The modeling of periodontitis in SD rats was completed. (C) The maxillae were analyzed by Micro-CT. (D) CEJ-ABC distance measured from micro-CT images. (E) 3D reconstructed and 2D images after 4 weeks of implantation. (F) BMP2 and OCN immunohistochemical image. (Black arrows indicate high expression areas).

To assess the therapeutic effect of Lap-Gel/H-E-Cu hydrogel in inhibiting alveolar bone defects associated with periodontitis, maxillary tissue samples were collected after 4 weeks and scanned using Micro-CT (Fig. 9A). As illustrated in Fig. 9B, a 0.2 mm arch wire was securely placed beneath the gums of the bilateral maxillary first molars to induce a periodontitis model. After ligation for 4 weeks, the periodontitis group exhibited significant alveolar bone loss in the interproximal region between the first and second molars, indicating the successful establishment of a chronic periodontitis rat model (Fig. 9C). The 2D and 3D images demonstrated the impact of different treatment modalities on the remodeling of the alveolar bone (Fig. 9E). In this study, the distance between the CEJ of the distal surface of the first molar and the alveolar bone crest (ABC) of the first and second molars was used as an indicator to assess the extent of alveolar bone loss [66]. After 4 weeks of treatment, the CEJ-ABC distance values were measured as follows: control group (0.501 ± 0.014 mm), periodontitis group (1.598 ± 0.031 mm), Lap-Gel group (1.307 ± 0.014 mm), and Lap-Gel/H-E-Cu group (0.747 ± 0.017 mm) (Fig. 9D). Compared to the periodontitis group, the Lap-Gel group demonstrated a smaller CEJ-ABC distance, indicating some preservation and recovery of alveolar bone height, while the Lap-Gel/H-E-Cu group showed the least difference from the control group, with minimal reduction in height, suggesting a good restoration of alveolar bone height. These results indicate that Lap-Gel/H-E-Cu hydrogel effectively promotes the recovery of alveolar bone volume in a periodontitis environment.

Additionally, immunohistochemical methods were employed to assess the activity of osteoblasts in the context of periodontitis, focusing on the markers OCN and BMP2. As depicted in Fig. 9F, the osteogenic factors OCN and BMP2 exhibited similar trends, with low expression in the periodontitis group, a moderate increase in the Lap-Gel group, and a significantly larger area of intense staining in the Lap-Gel/H-E-Cu group, where OCN and BMP2 expression was notably higher. These findings suggest that Lap-Gel/H-E-Cu hydrogel can effectively protect the periodontal ligament and alveolar bone while accelerating alveolar bone remodeling, presenting a promising approach for periodontal tissue repair.

### 3.9 Histopathological analysis

**Fig. 10:**
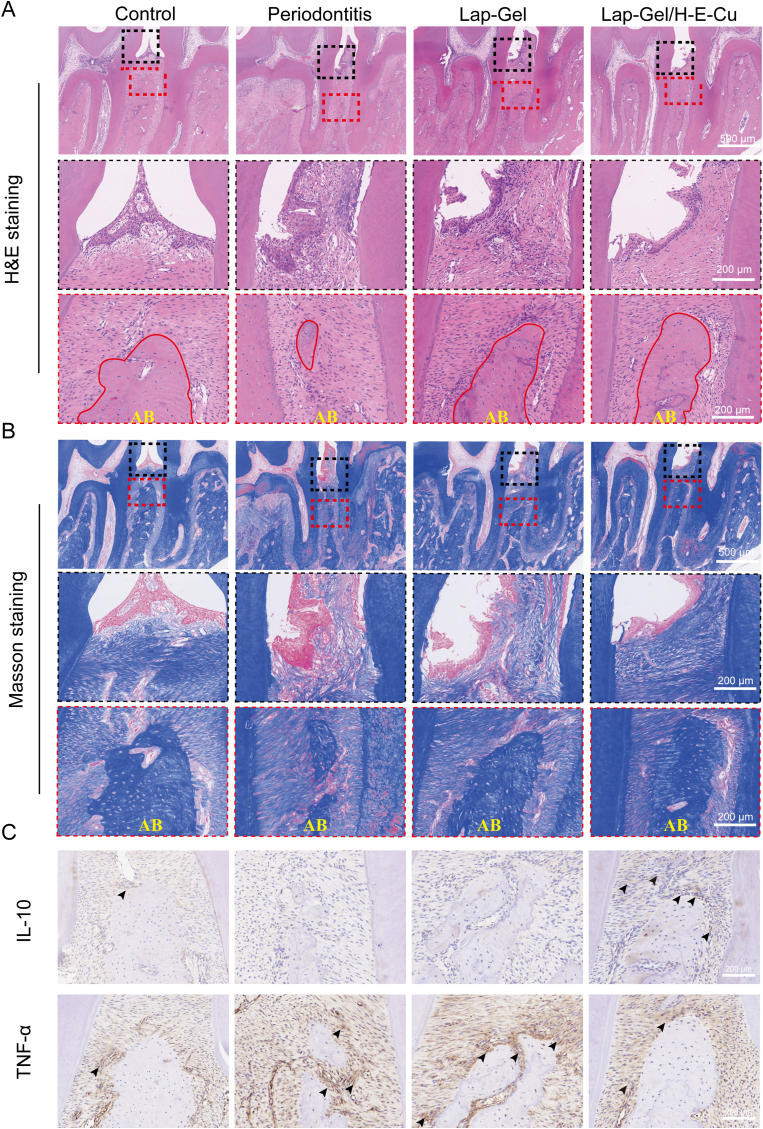
*In vivo* assessment of periodontal inflammation pathology in hydrogel-treated rats. (A) H&E staining of maxillae from the control, periodontitis, and two hydrogel treatment groups. (B) Masson's trichrome staining of maxillae across the control, periodontitis, and hydrogel treatment groups. (C) IL-10 and TNF-α stained images (Black arrows indicate high expression areas). AB: alveolar bone.

Chronic periodontitis damages periodontal soft and hard tissues, such as the gums, periodontal ligaments, and alveolar bone, eventually causing periodontal attachment loss [67,68]. To assess the protective effects of the Lap-Gel/H-E-Cu hydrogel on periodontal soft and hard tissues, periodontal tissue samples were obtained from the first and second molars and the maxilla, followed by H&E and Masson staining (Fig. 10A and B). After treatment, the control group maintained intact gingival morphology in the interproximal areas of the first and second molars, exhibiting a triangular shape with a complete epithelial structure. The epithelial layer was relatively thin, and no significant infiltration of inflammatory cells was observed beneath the epithelium. The alveolar bone appeared intact, with a rounded morphology, a certain thickness, and well-oriented and organized surrounding fibers. In contrast, the periodontitis group showed epithelial hyperplasia, with marked infiltration of inflammatory cells beneath the basal layer. The surface of the alveolar ridge exhibited significant bone resorption, osteoporosis, and surrounding inflammatory cell infiltration. The margins of the alveolar ridge became blade-like and thinned out, although there was a certain degree of recovery in the alveolar structure, indicating that Laponite has a favorable osteogenic effect. Compared to the periodontitis group, the Lap-Gel/H-E-Cu group exhibited only a small number of infiltrating inflammatory cells and demonstrated varying degrees of recovery in the shape and height of the alveolar bone. Additionally, no significant infiltration of inflammatory cells was noted beneath the epithelial basal layer, with dense fibers present, which positively influences the subsequent recovery of periodontal tissues. Disordered arrangement of peripheral fibers indicated a severe inflammatory response within the periodontal tissues. In the Lap-Gel group, the epithelial structure remained disordered, with obvious inflammatory cell infiltration beneath the basal layer; however, the alveolar bone height showed a certain degree of recovery while maintaining a relatively intact structure, further suggesting that Laponite possesses good osteogenic properties.

Simultaneously, this study employed immunohistochemical methods to analyze the changes in inflammatory markers such as TNF-α and IL-10. As shown in Fig. 10C, the expression of TNF-α was significantly higher in the periodontitis group compared to the normal control group. Similarly, the Lap-Gel group also exhibited elevated inflammatory expression. In the Lap-Gel/H-E-Cu group, however, the positive expression of inflammatory markers was relatively low. Conversely, IL-10 expression was significantly higher in the Lap-Gel/H-E-Cu group than in the periodontitis and Lap-Gel groups, demonstrating the hydrogel's strong anti-inflammatory activity *in vivo*.

## 4 Conclusion

In this study, we successfully designed an injectable hydrogel loaded with EGCG-Cu for the effective treatment of periodontitis. The Lap-Gel/H-E-Cu hydrogel exhibited excellent injectability and self-healing properties, allowing it to adapt to irregular tissue defects with ease. Additionally, it demonstrated a well-defined microporous structure, favorable mechanical properties, and optimal rheological behavior, ensuring its suitability as a versatile biomaterial for periodontal therapy. Furthermore, the hydrogel displayed outstanding biocompatibility and significantly enhanced the bioavailability of both EGCG and Cu^2+^, overcoming the inherent stability limitations of EGCG. *In vitro* studies revealed that Lap-Gel/H-E-Cu hydrogel effectively scavenged ROS, inhibited M1 macrophage polarization and enhanced M2 macrophage polarization, thereby mitigating the inflammatory cascade. Moreover, Cu^2+^ synergistically enhanced the antibacterial efficacy of EGCG, contributing to a more favorable periodontal microenvironment. Both *in vitro* and *in vivo* experiments confirmed the hydrogel's osteogenic potential, demonstrating its ability to facilitate bone tissue regeneration. By integrating immunomodulatory effects with osteogenesis-promoting properties, the Lap-Gel/H-E-Cu hydrogel significantly accelerated functional periodontal tissue repair. In conclusion, the Lap-Gel/H-E-Cu hydrogel, with its exceptional biocompatibility, controlled drug release, and dual therapeutic functionalities, holds great potential for promoting bone regeneration in periodontitis and repairing irregular and complex bone defects.

## CRediT authorship contribution statement

**Yajuan Hu:** Writing – original draft, Formal analysis, Data curation, Conceptualization. **Wei Xu:** Validation, Methodology, Formal analysis, Data curation. **Linghan Sun:** Methodology, Formal analysis. **Xuemin Ma:** Visualization, Software. **Peirong Zhou:** Software, Resources. **Chuankai Zhang:** Validation, Investigation. **Rui Cai:** Supervision, Methodology, Investigation. **Xia Wang:** Software, Investigation. **Hua Yang:** Investigation. **Gang Tao:** Writing – review & editing, Formal analysis. **Junliang Chen:** Writing – review & editing, Visualization, Funding acquisition, Formal analysis, Data curation. **Yun He:** Writing – review & editing, Visualization, Methodology, Investigation, Funding acquisition, Data curation, Conceptualization.

## Data availability

The data that support the findings of this study are available from the corresponding author upon reasonable request.

## Ethics approval and consent to participate

All animal experiments were approved by the animal ethics committee of Southwest Medical University (NO.: 20221214-005).

## Declaration of competing interest

The authors declare that they have no known competing financial interests or personal relationships that could have appeared to influence the work reported in this paper.

## Data Availability

Data will be made available on request.
