# Supplementary material for: Multifunctional injectable hydrogel incorporating EGCG-Cu complexes for synergistic antibacterial, immunomodulatory, and osteogenic therapy in periodontitis
Source: Mater Today Bio. 2025 May 28;32:101907. doi: 10.1016/j.mtbio.2025.101907 (PMC12164233; doi:10.1016/j.mtbio.2025.101907)
Supplement: Multimedia component 1 [file mmc1.docx]

**Multifunctional Injectable Hydrogel Incorporating EGCG-Cu Complexes for Synergistic Antibacterial, Immunomodulatory, and Osteogenic Therapy in Periodontitis**

Yajuan Hu^1, 2, 3, *^, Wei Xu^1, 2, 3, *^, Linghan Sun^1, 2, 3^, Xuemin Ma^1, 2, 3^, Peirong Zhou^1, 2, 3^, Chuankai Zhang^1, 2, 3^, Rui Cai^1, 3^, Xia Wang^1, 3^, Hua Yang^5^, Gang Tao^1, 3 #^，Junliang Chen^1, 2, 3, 4, #^, Yun He^1, 2, 3 #^

**Author details**

^1^ Luzhou Key Laboratory of Oral & Maxillofacial Reconstruction and Regeneration, The Affiliated Stomatological Hospital, Southwest Medical University, Luzhou 646000, China

^2^ Department of Oral and Maxillofacial Surgery, The Affiliated Stomatological Hospital, Southwest Medical University, Luzhou 646000, China

^3^ Institute of Stomatology, Southwest Medical University, Luzhou 646000, China

^4^ Department of General Dentistry, The Affiliated Stomatological Hospital, Southwest Medical University, Luzhou 646000, China

^5^ Department of Oral and Maxillofacial Surgery, The Deyang Stomatological Hospital, Deyang 618000, China

^*^ These authors contribute equally to this study.

^#^ Correspondence:

Gang Tao, Luzhou Key Laboratory of Oral & Maxillofacial Reconstruction and Regeneration, The Affiliated Stomatological Hospital, Southwest Medical University, Luzhou 646000, China, E-mail: taogang@swmu.edu.cn

Junliang Chen, Luzhou Key Laboratory of Oral & Maxillofacial Reconstruction and Regeneration, The Affiliated Stomatological Hospital, Southwest Medical University, Luzhou 646000, China, Email: [cjlhyy@163.com](mailto:cjlhyy@163.com)

Yun He, Luzhou Key Laboratory of Oral & Maxillofacial Reconstruction and Regeneration, The Affiliated Stomatological Hospital, Southwest Medical University, Luzhou 646000, China, Email: heyundaidai@163.com

Figure S1. Cumulative release profiles of Cu^2+^ in 90 h.

Figure S2. Cumulative release profiles of EGCG in 90 h.


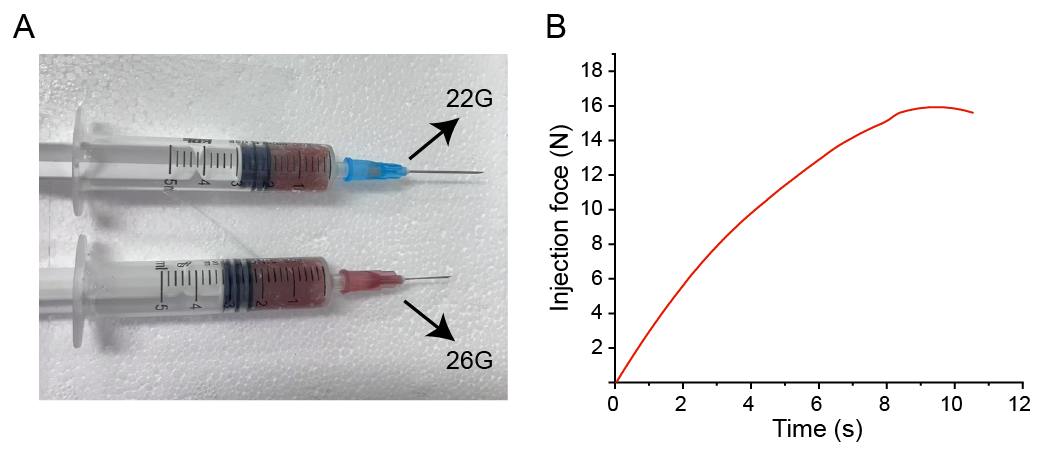


Figure S3 Injection force of the Lap-Gel/H-E-Cu hydrogel. (A) Photographs of Lap-Gel hydrogel loaded into 5 mL syringes fitted with clinical needles of different gauges: 22G (top) and 26G (bottom). (B) Injection force profile of the hydrogel extruded through a 22G needle measured under constant-speed compression (2 mL/min), showing a peak force of approximately 16 N.


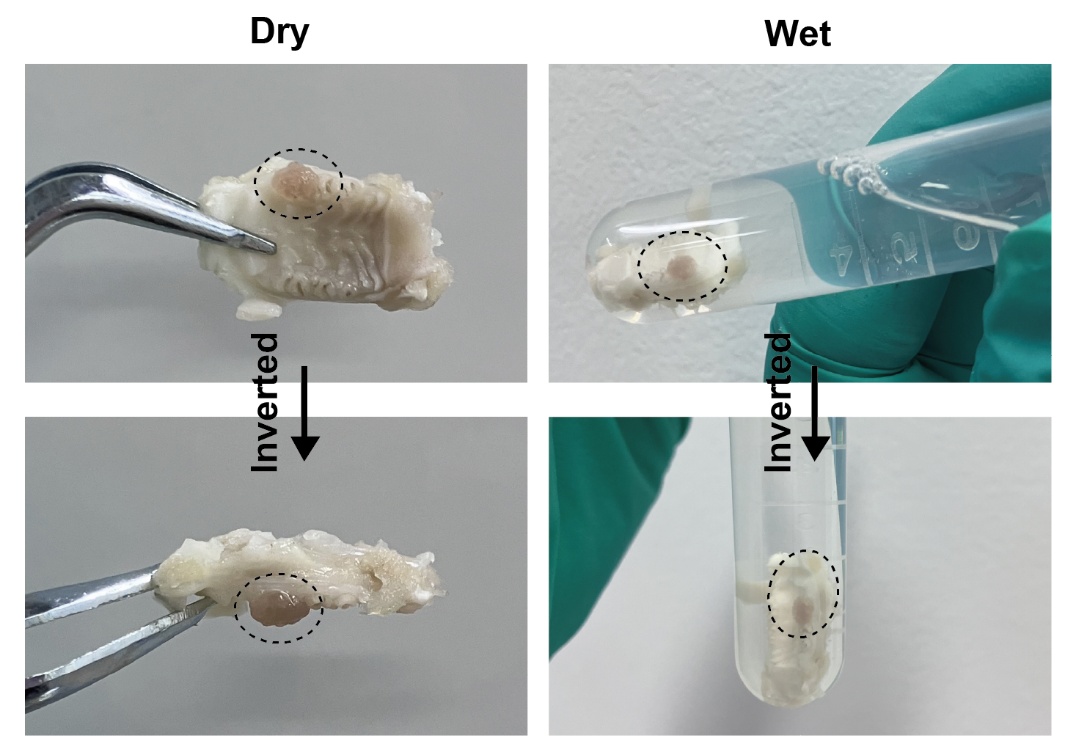


Figure S4. Retention of the EGCG-Cu-loaded hydrogel on excised rat maxillary periodontal tissue under dry (left) and wet (PBS-immersed, right) conditions.

Figure S5. The weight remaining ratio of Lap-Gel/H-E-Cu hydrogel in PBS.
